# A negative storage model for precise but compact storage of genetic variation data

**DOI:** 10.1093/database/baz158

**Published:** 2020-04-15

**Authors:** Guillermo Gonzalez-Calderon, Ruizheng Liu, Rodrigo Carvajal, Jamie K Teer

**Affiliations:** 1 Biostatistics and Bioinformatics Shared Resource, H. Lee Moffitt Cancer Center and Research Institute, 12902 Magnolia Dr., Tampa, FL, 33912, USA; 2 Department of Biostatistics and Bioinformatics, H. Lee Moffitt Cancer Center and Research Institute, 12902 Magnolia Dr., Tampa, FL, 33912, USA

**Keywords:** data storage, next-generation sequencing, genetic variation

## Abstract

Falling sequencing costs and large initiatives are resulting in increasing amounts of data available for investigator use. However, there are informatics challenges in being able to access genomic data. Performance and storage are well-appreciated issues, but precision is critical for meaningful analysis and interpretation of genomic data. There is an inherent accuracy vs. performance trade-off with existing solutions. The most common approach (Variant-only Storage Model, VOSM) stores only variant data. Systems must therefore assume that everything not variant is reference, sacrificing precision and potentially accuracy. A more complete model (Full Storage Model, FSM) would store the state of every base (variant, reference and missing) in the genome thereby sacrificing performance. A compressed variation of the FSM can store the state of contiguous regions of the genome as blocks (Block Storage Model, BLSM), much like the file-based gVCF model. We propose a novel approach by which this state is encoded such that both performance and accuracy are maintained. The Negative Storage Model (NSM) can store and retrieve precise genomic state from different sequencing sources, including clinical and whole exome sequencing panels. Reduced storage requirements are achieved by storing only the variant and missing states and inferring the reference state. We evaluate the performance characteristics of FSM, BLSM and NSM and demonstrate dramatic improvements in storage and performance using the NSM approach.

## Introduction

Massively parallel sequencing results represent the latest emergence of ‘big data’ in the life sciences. Although many of the technical and analytical challenges and issues are no different from those that came before (for example, in microarray or capillary sequencing data), massively parallel sequencing data represents a large increase in the amount of information (around 10 000× increase in total bases stored by the Sequence Read Archive since 2009 https://trace.ncbi.nlm.nih.gov/Traces/sra/sra.cgi? accessed 17 December 2018). Many methods and tools have been developed for compression and storage of sequence reads (i.e. SAM/BAM/CRAM (1)) and variation data (VCF, BCF, MAF, tabix (2)). Variation data is of particular interest given the desire to analyze, query and integrate the data of many individual samples. Storage of sequencing data from many individual samples is certainly possible using structured text files, and many programs are able to analyze such multi-sample files (VarSifter (3), SVA (4), GEM.app (5)). However, as more and more samples are sequenced, database storage technologies are increasingly being leveraged to store genetic data. This offers distinct advantages to data storage: structured query language (SQL) approaches and strategies have been applied to numerous other fields with great success, in part due to a common query language. Large-scale visualization web applications like the UCSC Genome Browser (6), cBioPortal (7), MEXPRESS (8) and COSMIC (9) have leveraged database technology to display summary genomics information rapidly. In addition to classic SQL models, new database philosophies have been developed to focus more on the ability to store larger amounts of data in part by dispensing with some of the strict rules of data organization and de-duplication (‘normalization’) that characterize SQL databases. Such systems, often termed ‘NoSQL’ databases, have proven their worth via extensive use in internet content storage systems used in current popular social media products. They have also been shown to offer improved performance for genomic annotation storage (10). Many platforms and interacting frameworks have already been developed to store, query and present data in visually appealing graphical user interfaces. Many forms of associated information (for example, patients’ clinical information) may already be stored in such systems. The addition of genomic variation data therefore allows for integrated queries that enable improved understanding of the relationship between genotype and phenotype.

Even though database systems are designed for large amounts of data, it is unclear to what extent they will scale when faced with genome level information across many thousands of samples. For example, given a 3 billion base pair human genome, 10 000 patients would involve 30 trillion rows of data. This has been initially addressed by restricting the regions of the genome undergoing sequencing. Although sequencing costs have dramatically decreased (11), further cost savings have come from targeted capture before sequencing, such as is commonly applied via a variety of whole exome sequencing (WES) commercial kits. This reduces the number of potential variants proportionally to the capture target (i.e. WES results in ~1% the data of whole genome). Many home-grown variation database solutions have further addressed this issue by only storing genetic variations compared to a reference genome. Given a 3 billion base pair human genome, a per genome variation rate of 0.13–0.16% per genome (12), and a much lower somatic mutation rate in cancer (13), storing only inherited variants would result in a >99% reduction in information stored.

**Figure 1:**
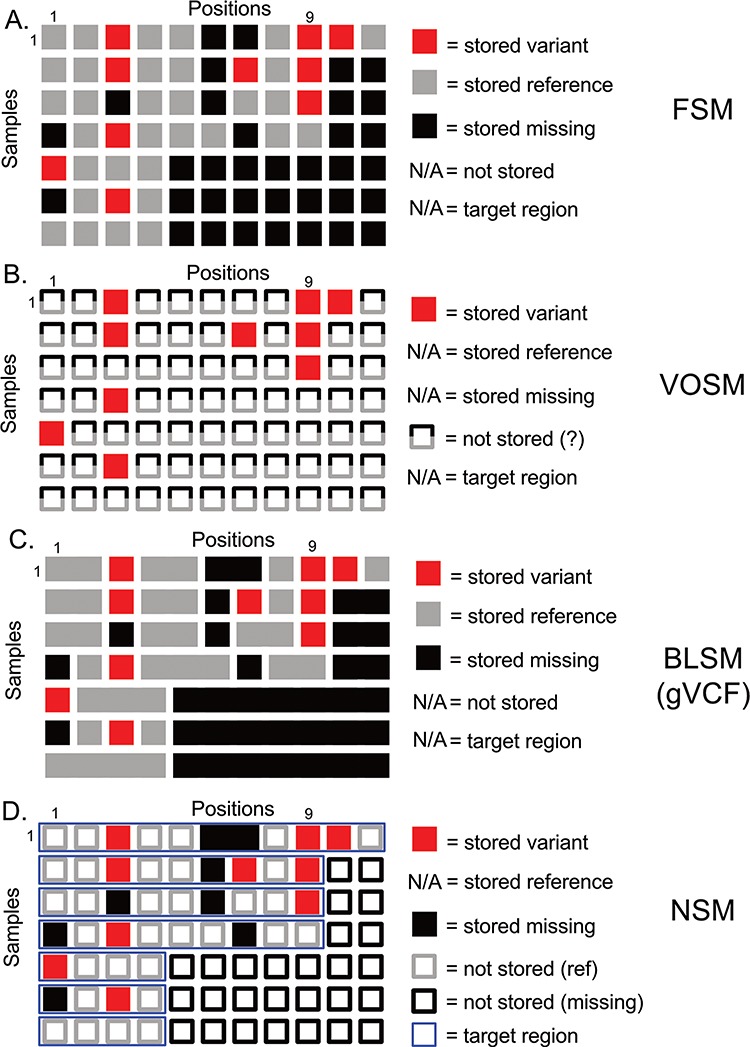
Overview of genetic variation storage models. This example shows the same 7 samples and 11 positions using (**A)** Full Storage Model, (**B**) Variant Only Storage Model, (**C**) Block Storage Model and (**D**) NSM. The features and information stored in each model are listed to the right. FSM: Full Storage Model, VOSM: Variant Only Storage Model, BLSM: Block Storage Model, NSM: Negative Storage Model.

However, storing only genomic variants results in a potential loss of precision. The lack of a stored variant at a given position is usually inferred to mean that the sample has a reference genotype. However, given that sequencing is not 100% sensitive to detect variants, there is also the possibility of a missing or low quality result that should NOT be assumed to be reference. An even greater challenge is often seen within institutions, including our own. When many different capture kits are being used in both research and clinical settings, the absence of a variant could represent a different type of missing data: the untested position. Therefore, there is a distinct need for a genetic variation storage strategy that reduces that amount of stored information but preserves the precision to know whether a given position in a sample was reference, variant or missing. We have developed a data storage approach, the Negative Storage Model (NSM), by inverting the typical paradigm of storing only what is known. This model stores tested regions, known variants within those regions and, counter-intuitively, those positions that were missing in the dataset.

## Introduction: storing genetic variation data

Genetic variation data defines locations in the genome that distinguish individuals from one another and their genetic code (genotypes) at these positions. The most straightforward method of storing this information is to imagine a matrix with one column per genome position, and one row per sample. We term this model the Full Storage Model (FSM). In a simplistic example, a cohort of 7 human samples with 11 positions of interest could be represented by a 7 patient × 11 position matrix (Figure 1). In practice, the matrix could include thousands of samples and 3 095 693 981 positions. Each element of the matrix stores the state of a position in a given sample: reference (or non-variant), variant (or mutated) and missing (or insufficient data) (Figure 1a). Getting the exact state of a position-sample from this model is very easy. Consider Position 9 in Figure 1a. We can easily determine that four of seven samples had information (three out of seven were missing at that position), and three out of four samples with data had a variant. However, this storage matrix is quite large, potentially leading to poor performance characteristics. Since we know that much of the human genome is non-varying, many positions will not hold differentiating information.

A second option focuses more on the variants themselves and indeed stores nothing else (Figure 1b). However, we lose all information about non-variant positions. We term this model the Variant-Only Storage Model (VOSM). If sequencers were perfect, and we never had missing data, such a system would maintain precision as we could safely assume everything not stored was non-variant. However, sequencing instruments have error rates, targeted capture sequencing results in incompletely covered genomes and depth-of-coverage variations result in positions with insufficient information to conclude a position is non-variant. For example, Position 9 shows three out of seven samples with a variant, and the remaining four out of seven are assumed to be non-variant. However, we know the non-variant assumption to be false as the last three samples are missing information about that position. Although the storage efficiency of a variant-only model is high, the loss of precision and potential for highly inaccurate results is a significant risk.

A third option retains the precision of the FSM, but collapses individual positions into contiguous blocks of common information state (reference, variant or missing). The Block Storage Model (BLSM) is most commonly seen in gVCF file output as part of certain genetic variant identification tools, including the Genome Analysis Toolkit “HaplotypeCaller” module (14). This model, like the FSM, accounts for every base in the genome. However, a level of compression is achieved by combining positions of common state together in a single block (Figure 1c). For example, an unbroken group of reference bases can be joined together as one data entry. This offers precision, and a level of compression, but still requires storing information outside of targeted regions.

Current genetic variation storage models have distinct weaknesses. Although the Full Storage Model offers high precision, it requires a large amount of storage space, and we show that performance can be poor. The Variant-Only Storage Model allows for improved performance and is therefore widely used. However, it can be considered a ‘lossy’ compression method, and the loss of precise information for non-variant positions can result in inaccurate results. The risks of inaccuracy are highest when considering large datasets of distinct capture kits, where the targeted regions and sequencing technologies may be different across samples. For example, an institution wishing to store sequencing data generated by several large but different research projects as well as more focused clinical sequencing panels would have distinct target genes and coverage profiles over those genes, resulting in a risk of inaccurate assessment of mutation frequencies across the sample set.

## Results: NSM

We propose a storage model (Figure 1d) for genetic variation data where we store not only what is known (genetic variants) but also what is unknown (missing positions). This information is combined with knowledge of the regions that were actually tested (the target region) to infer the reference state. From studies of human genetic variation and tumor mutations, we know that the majority of positions in the genome are invariant between individuals. Therefore, we leverage that knowledge to save storage by inferring this state instead of storing it. We restrict this assumption to those positions that were actually tested (based on definitions of a genomic capture target region, if appropriate). Any positions outside a sample’s target region are inferred to be missing (Figure 1d, empty black squares). We store as variants (Figure 1d, filled red squares) those differences from the human reference genome along with their associated contextual information (amino acid change, frequency in public databases, etc). We store as missing (Figure 1d, filled black squares) those positions that had insufficient data to determine whether a variant is present. Since the quality of sequencing data is generally high, only a minority of positions is expected to be ‘missing’ based on absence, insufficient depth of coverage or other issues resulting in a low confidence variant determination. In contrast, most non-variant positions (Figure 1d, empty grey squares) are high-confidence reference alleles. Therefore, our assumptions regarding ‘reference’ without actually storing this information are more than simple assumption: we have specifically identified those positions that violate this assumption and stored them as missing data. This strategy enables precise knowledge of the exact state of a given position, be it variant, non-variant or missing, while reducing the amount of information actually stored.

Consider the following example based on Position 1 in Figure 1d. All samples include Position 1 in the target region (thin blue horizontal boxes), so we should have information for all samples. Samples 1–3 and 7 are non-variant, and so nothing is stored—we will infer these positions to be non-variant. Sample 5 has a variant, and the variant information is stored. Samples 4 and 6 are missing data and are therefore neither variant nor reference. We store a ‘missing’ value here. So, we are able to determine that five out of seven samples have information at Position 1, and one out of five have a variant. In our earlier example, we have stored variants at Position 9 for Samples 1–3. For Sample 4, we know that Position 9 is in the target region and, as nothing is stored, will infer that it is reference. For Samples 5–7, we know Position 9 is not included in the target region, which will therefore be inferred to be missing. We can infer precise information to know that three out of four samples are variant.

**Figure 2:**
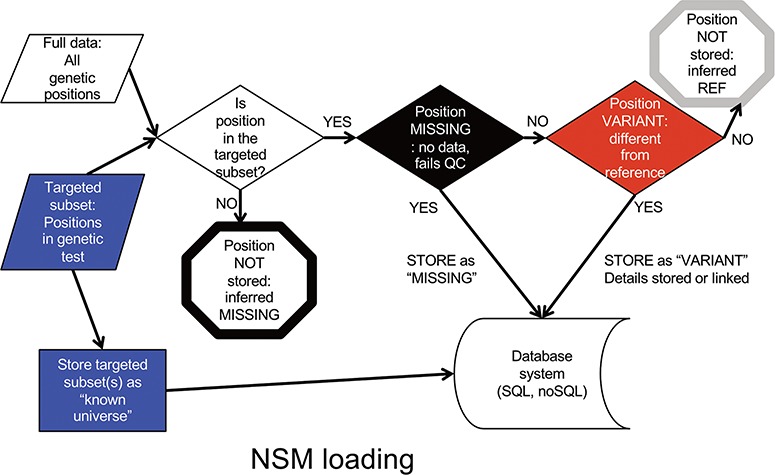
NSM data loading process. The process for determining what information should actually be stored in the database.

Data is input into the NSM in a multistep process (Figure 2). First, a targeted subset of positions is defined. This subset includes those positions targeted by a particular targeted capture kit used in a sequencing experiment (e.g. Whole Exome, focused clinical panel, custom gene panel). Then, all positions within the targeted region are assessed for quality. If there is insufficient data to have accurately determined the genotype at a position, the position is considered ‘missing’, and the value of ‘missing’ is stored at that position for that particular sample. If data quality is sufficient, and the genotype is a variant compared to the reference, the variant and its associated contextual information are stored. If data quality is sufficient, and the genotype matches the reference, it is not stored, but later inferred to be ‘reference’.

**Figure 3:**
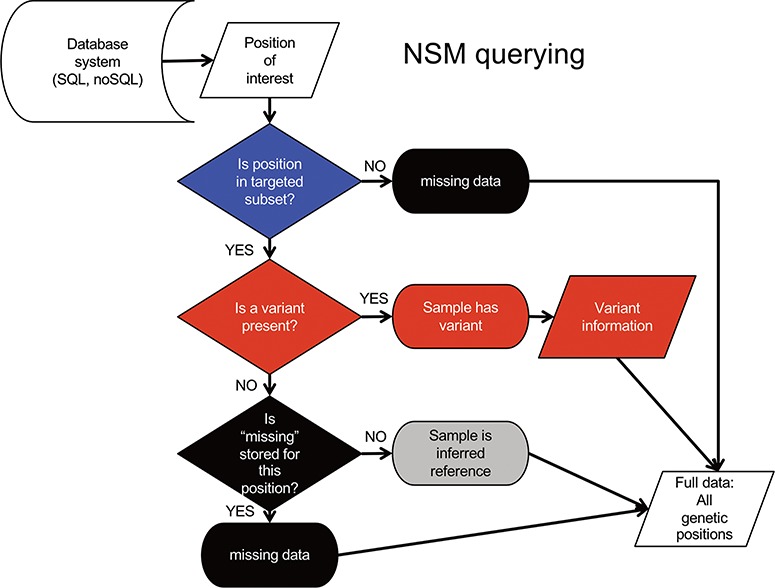
NSM data querying process. The process for extracting precise information for a given position based on the model and information stored in the database.

Querying data from the NSM follows an inverse sequence of events (Figure 3). First, it is determined whether or not a query position is in the targeted subset. If the query position is NOT within the target region, a ‘missing’ value is returned as there is no knowledge of positions outside the target region. If the query position is within that sample’s target region, a check for ‘variant’ status is performed. If a variant is present, the associated variant information is returned. Otherwise, a check for ‘missing’ status is performed. If a ‘missing’ value is present, the ‘missing’ status is returned. If no values are present, a value of ‘reference’ is returned, as it can be inferred from the loading procedure that the absence of ‘missing’ indicates sufficient data quality at the position, and the absence of a variant indicates the genotype is reference.

## Results: performance

The NSM can be implemented in standard SQL databases using custom schema and queries. To demonstrate performance, we compare our NSM to the FSM, where all pieces of information (variant, reference, missing) are stored, and to the BLSM, where all pieces of information are stored compressed as blocks of genomic regions. VOSM is not tested as the potential for inaccuracy excludes it from consideration based on our requirements. NSM and FSM schemas were implemented in SQL databases (MySQL, Oracle) and a NoSQL database (MongoDB). A BLSM schema was implemented in MongoDB. To test the model, we stored the mutation information from 367 tumor samples from the TCGA SKCM WES dataset downloaded from tcga-data.nci.nih.gov (March 2016). Coverage files were downloaded from https://www.synapse.org/#!Synapse:syn1695394. In this proof of concept, the region of interest is defined as the padded coding exons of RefSeq genes.

We first evaluated the storage requirements of both approaches. The FSM stored a value of ‘mutated’ (with associated annotation information), ‘unmutated’, or ‘missing’. A known target region of 43 022 725 bases (including 25 bp on each side of an exon) was stored for each sample, resulting in a total of 15.8 billion data elements (Table 1). The BLSM stored similar values for contiguous blocks of sequence, resulting in only 164.9 million elements. The NSM stored even fewer elements: across the 43 million targeted base pairs, only mutations and missing data were stored. The majority of positions, being neither mutations nor missing, were inferred to be the same as reference. The total of 140.7 million elements was much lower than the FSM, and slightly lower than the BLSM, but maintained the precision necessary for rigorous downstream analyses. Unsurprisingly with many fewer data elements stored, database size for NSM was 0.7–0.8% of the full storage model (NSM: 6.3–13.8 GB; BLSM: 14.6 GB; FSM: 876–1665 GB). NSM storage size was 43–95% of the BLSM, indicating both models compress precise variant information well, with NSM being somewhat better. These sizes included indexes generated to support subsequent query testing.

The load times associated with NSM were next compared to FSM and BLSM. Given large amounts of data to be stored, the time needed to load data can be significant. NSM load times (including indexes) were much faster than FSM, ranging from 0.8–2 h in total, while FSM ranged from 148–1296 h (6–54 days). NSM was also much faster than BLSM: 453 h (19 days) with the majority of time being spent on pre-calculating the block structure. Load times, particularly for the FSM databases, were optimized by creating indexes after data loads.

The NSM resulted in decreased data storage, but queries were more complex. Query execution time was tested using four different use cases (Box 1). The NSM query time (MongoDB implementation) was often faster across all test cases (Table 1), with the notable exception of the MongoDB implementation of the FSM, which was fastest at all queries. BLSM query performance was somewhat slower than the NSM MongoDB implementation except for Query 4. Query outputs from the different strategies were identical, demonstrating the precision of the NSM.

Box 1 Queries used as test cases for evaluation of storage model performance

**1. Return sample status at a position**

- INPUT: chromosome , position
- OUTPUT: status (ref, alt, missing) for all samples

**2. Return sample status for a given mutation (gene, amino acid change)**

- INPUT: gene, amino acid change
- OUTPUT: status for all examples

**3. Return sample status at all mutations in a gene**

- INPUT: gene
- OUTPUT: status for all samples at all mutations

**4. Return sample coverage across a gene**

- INPUT: gene
- OUTPUT: fraction bases with high quality data per sample

**Table 1:** Performance metrics of genetic variant storage models

Approach	Load time	Use case 1	Use case 2	Use case 3	Use case 4	DB size (GB)	DB record (rows)
**FSM** MySQL^1^	216 h	600–4000	600–4000	1.1 M–10.5 M	216	1100	15.8B
**FSM** Oracle^1^	1296 h	~1	~1	2628	33	1665	“
**FSM** MongoDB^1^	148 h	0.002	0.002	0.746	0.009	876	“
**BLSM** MongoDB^1^	453 h	5.5	1.9	87.1	0.1	14.6	164.9 M
**NSM** MySQL^2^	2 h	13.2	12.1	635	0.01	13.8	140.7 M
**NSM** MongoDB^1^	0.8 h	1.6	0.7	43.9	0.361	6.3	“

Time in seconds, unless noted

^1^16× Xeon E7-4480, 256 GB RAM, SSD

^2^4× Xeon ES-2650, 64 GB RAM, SSD

## Discussion

We have proposed a novel model for the precise storage of genetic variation data. This model accounts for the state (reference, variant or missing) of every position in the genome without having to store each piece of information. Variation and missing data are stored, and reference data (the vast majority of information in sequencing experiments) is inferred based on targeted genomic regions and absence of variation or missingness. This results in the ability to precisely know the status of a given position across samples. We have outlined the algorithms for loading and extracting data from the model we have termed the NSM.

We have performed proof-of-principle testing on somatic mutation data from 367 TCGA Skin Cutaneous Melanoma samples. The NSM was compared to a full storage model (FSM), where every coding base is stored with its state, and to a Block Storage Model (BLSM), where the information for individual bases is compressed into blocks as in the gVCF file format. We found that although the NSM required more complex queries, performance was similar to or better than FSM. This is likely due to the smaller amount of data to be traversed by a given query. NSM queries were slightly faster than BLSM, suggesting that while both methods of compression are effective, NSM may have a performance advantage. We also found that the MongoDB NoSQL database is a powerful tool for querying large databases and was effective for all three approaches. This required the use of indexes; without indexes, MongoDB performance using the NSM was much worse (Supplemental Table 1), suggesting that indexing strategies will remain a critical part of NoSQL database tuning. We observed that data loading was much faster with NSM. NSM required hours, whereas FSM required days (up to 54 days for the Oracle example). FSM loading times did not include time spent rerunning the load due to a hardware issue that caused an initial attempt to fail before completion. BLSM also required a longer time to load (19 days) due to the need to precompute the block structure. Load times for each approach could likely be decreased with additional model-specific tuning.

Although queries in the FSM are simpler, the larger amount of data created many challenges. Numerous errors were encountered during data loading and querying, particularly using the Oracle and MySQL implementations. Any interaction with this data strategy was qualitatively much more fragile due to resource exhaustion, hardware vulnerabilities and other typical error modes of computation.

Querying the NSM required more effort than the full storage model due to additional requirements (Figure 3). However, range-style queries (query position is >= start and <= end) were effective in determining whether a given position was included in the target region. These range-style queries were also well-suited to the BLSM given its block structure, but were distinct from those used in NSM. We envision an API would be important to separate the details of given database queries from other layers (including visualization). It is important to note that NSM is a storage model. We implemented NSM as proof of concept using several database systems. As with any technology, increasing resources are needed to handle larger amounts of data, and an approach suitable for thousands of samples may be overkill for smaller use cases. Going forward, we are exploring alternate implementations to allow scaling from small datasets (using file-based or in-memory databases) to larger (using dedicated database servers or clusters).

In summary, we have described strategies for storage of genetic variation data: the Variant Only Storage Model, Full Storage Model, Block Storage Model and NSM. The VOSM does not support full precision of genomic variants to be stored. The FSM does provide this precision but with significant performance and storage limitations. The BLSM improves performance and storage requirements while maintaining precision, although there is an initial cost to calculate the block boundaries and information. The NSM is a novel approach to this particular large data problem and leverages the observation that the human genome is largely invariant across individuals. Instead of storing everything we know, NSM stores the less common variations and missing data. Leveraging what we should know (the targeted region for Whole Exome and other targeted sequencing panels), we can then infer those regions that have a reference genotype, given the absence of a stored variant or missing position. NSM has been used by our group as a ‘staging’ system to bring a variety of DNA sequencing experiment types together into a single system. Once the data are precisely stored, they can be exported in a variety of formats (including the precise gVCF file). In this way, we have harmonized different data types together for eventual analysis. Although a Full Storage or Block Storage Model can be used for this purpose, we introduce the NSM as a more efficient way to store ever-increasing amounts of genetic variation data.

## Methods

Proof of concept schemas for NSM and FSM were implemented in MySQL (v5.7, engine:InnoDB), Oracle (12C) and MongoDB (v4, engine: Wired Tiger). Proof-of-concept BLSM was implemented in MongoDB (v4, engine: Wired Tiger). We used 367 tumor samples from the TCGA SKCM WES dataset as a test cohort. The Level 3 somatic mutations were downloaded from tcga-data.nci.nih.gov (March 2016). Coverage files were downloaded from https://www.synapse.org/#!Synapse:syn1695394. The region of interest is defined as the coding exons of RefSeq genes plus 25 bp padding, downloaded from the UCSC Genome Browser. All models started with the same mutation dataset and coverage. Performance was evaluated on one of the following servers: 4× Xeon ES-2650, 64GB RAM, SSD storage; 16× Xeon E7-4480, 256GB RAM, SSD storage. Database caches were flushed after each query to ensure consistent measurements.

The code, data and documentation for the storage models are available at the following link: http://lab.moffitt.org/teer/genetic-variation-database-storage-models/.

## Supplementary Material

Supp_Table_1_baz158
