# Supplementary figures and images for: A negative storage model for precise but compact storage of genetic variation data
Source: Database (Oxford). 2020 Apr 15;2020:baz158. doi: 10.1093/database/baz158 (PMC7157186; doi:10.1093/database/baz158)

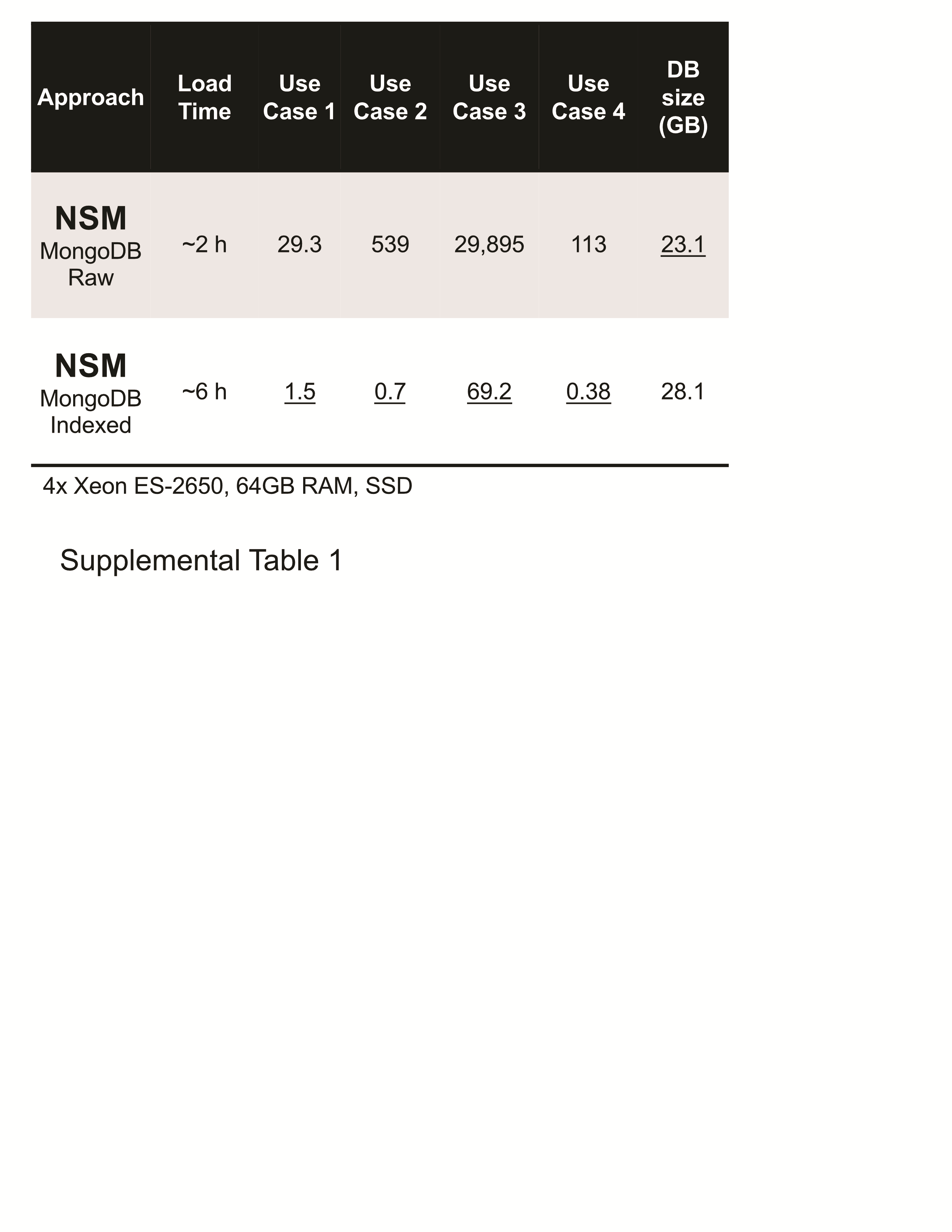

Supplement: Supp_Table_1_baz158 [file supp_table_1_baz158.png]
